# Looking at IGF-1 through the hourglass

**DOI:** 10.18632/aging.204257

**Published:** 2022-08-25

**Authors:** William B. Zhang, Sofiya Milman

**Affiliations:** 1 Physician-Scientist Development Program, Department of Pathology, University of Chicago, Chicago, IL 60637, USA; 2 Department of Medicine and Genetics, Institute for Aging Research, Albert Einstein College of Medicine, Bronx 10461, NY

**Keywords:** IGF-1, aging, growth hormone, mortality, morbidity

The role of insulin-like growth factor 1 (IGF-1) in human aging has been hotly debated. IGF-1, a central regulator of growth, plays a critical function in development and energy investment. For instance, the absence of functional IGF-1 or IGF-1 receptors in the central nervous system leads to severe developmental abnormalities, whereas reduction in circulating IGF-1 level, which is regulated by growth hormone (GH), results in small whole-body size [1]. In contrast, during aging, circulating IGF-1 may become dispensable or even detrimental. In fact, reduced long-term GH/IGF-1 improves the lifespan and health of numerous model organisms, including nematode worms, fruit flies, and mice. Despite strong evidence for beneficial effects of diminished GH/IGF-1 signaling in aging models, human studies investigating IGF-1’s role in aging and age-associated diseases have been discrepant and until recently it has not been possible to reconcile the inconsistent results.

Extreme phenotypes in humans can shed some light on the controversy. Acromegaly and Laron dwarfism provide examples of medical conditions resulting from extreme excess or insufficiency of IGF-1, respectively. The excess GH and IGF-1 of acromegaly cause a variety of harmful effects, including hypertension, diabetes, cardiac dysfunction, colonic polyps, and arthritis; all of these can be classified as conditions that become more prevalent with aging. In contrast, the attenuated IGF-1 signaling that results from defective GH receptors of Laron dwarves seems to confer protection from cancer, stroke, and diabetes while increasing obesity and auditory deficits. Thus, it appears that exceptionally high circulating levels of IGF-1 are generally harmful, while extremely low IGF-1 signaling results in a combination of benefits and harms.

However, direct study of cohorts with more modest variability in circulating IGF-1 levels has yielded conflicting results. While some previous studies, including ours, have shown a positive association between high IGF-1 and adverse outcomes in respect to mortality and age-related co-morbidities [2,3], others have found the opposite effect or no associations [4]. These prior studies were generally conducted in modestly-sized samples and varied significantly in the basic demographics of the participants. Nonetheless, one pattern did begin to emerge from these diverse studies: the beneficial effects of lower IGF-1 were most apparent among the oldest age groups. Thus, it became evident that in order to resolve the controversy, it was necessary to study a large cohort with a broad age range.

In an effort to fulfill that mission, we leveraged the scale of the UK Biobank to examine the interactions between serum IGF-1 levels and participant demographics more explicitly [5]. We examined the association between serum IGF-1 and a panel of age-associated outcomes, including vascular disease, diabetes, dementia, osteoporosis, cancer, and mortality. By studying a large cohort of hundreds of thousands of participants, the study had sufficient statistical power to investigate how IGF-1 differentially associated with outcomes in younger vs. older participants, as well as how these associations changed over the full dynamic range of IGF-1 serum values.

**Figure 1:**
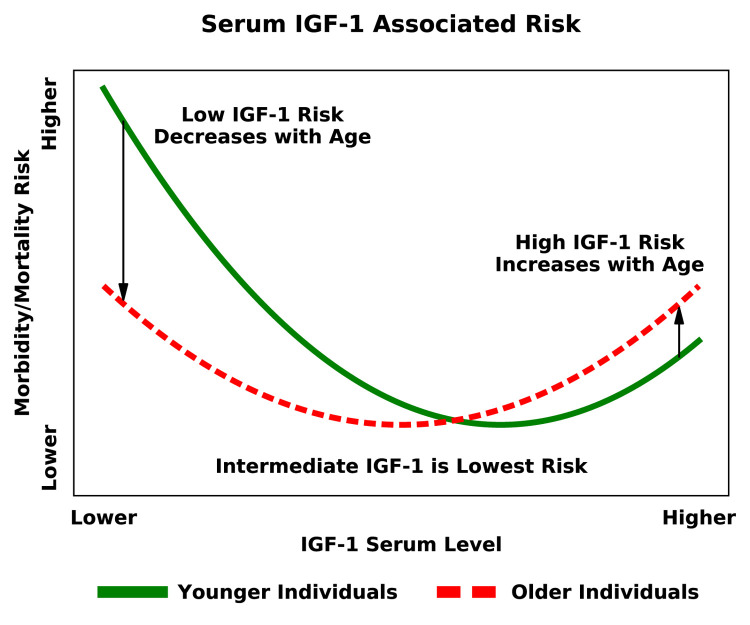
Risk of mortality and morbidity associated with serum IGF-1 levels, stratified by age groups [5].

That study produced a unifying framework by which the apparently discrepant results in previous studies can be reconciled: IGF-1 is a nonlinear predictor of risk and interacts with age to modify risk for a variety of clinical events. Specifically, IGF-1 is mostly associated with protection from disease in younger individuals; conversely, it is associated with increased morbidity and mortality in older individuals. In addition, the disease risk associated with serum IGF-1 levels is generally U-shaped [6]: Across all age groups studied, both the highest and lowest serum IGF-1 levels are associated with detrimental outcomes when compared to more moderate levels (Figure 1).

As a whole, our recent study suggests a dual role for IGF-1 in development and aging. While it is required for regulated development and growth in younger individuals, it may impede repair and somatic maintenance in older individuals through inhibition of autophagy. This is supported by human cohorts with exceptional longevity, who have been shown to be enriched in variants in genes in the IGF-1 pathway [7]. Some of these genetic variants may result in decreased IGF-1 signaling and may therefore be partially responsible for the longevity phenotype. Further, experimental attenuation of IGF-1 signaling in older mice has been shown to increase female median lifespan and health-span [8].

While high IGF-1 may be causally implicated in accelerated aging and increased disease burden, the mechanistic connection between low IGF-1 and clinical risk is less well-established. One possible explanation is that IGF-1 levels decrease with age, so that higher IGF-1 can be considered a biomarker for youth. Therefore, it is possible that some individuals with low IGF-1 currently may have actually experienced accelerated aging in the past. Another possibility is that low IGF-1 is known to occur in patients with either chronic or acute illness, and may sometimes be a biomarker for underlying latent disease processes. As a result, low IGF-1 may be caused by undetected pre-existing disease (“reverse causation”), rather than low IGF-1 itself causing future disease. Ongoing longitudinal studies with repeated measurements of IGF-1 may contribute to exploring these possibilities.

These latest results may help resolve the controversy over whether higher serum IGF-1 levels are associated with benefit or harm in humans. Previous studies reached simpler and often opposing conclusions about high serum IGF-1 being either associated with generally worse outcomes or generally better outcomes. Our more comprehensive study suggests that those earlier results may have drawn from segments of the population (e.g. younger or older individuals) for whom the beneficial or harmful associations of IGF-1 are more pronounced. These findings underscore the importance of looking at the role of IGF-1 in health-span and lifespan through the lens of aging.
